# New insights into enlarged parietal foramina: an anatomical, radiological, and histological study

**DOI:** 10.1007/s00381-026-07197-w

**Published:** 2026-03-15

**Authors:** Laurel A. Seltzer, Brianna L. Hines, Zachary S. Weisberg, David Ezra, Joseph Lockwood, Hotaka Kawai, Noritaka Komune, Joe Iwanaga, Aaron S. Dumont, R. Shane Tubbs

**Affiliations:** 1 Tulane University School of Medicine, New Orleans, LA USA; 2 School of Nursing Sciences, The Academic College of Tel Aviv Jaffa, Tel Aviv Jaffa, Israel; 3 Department of Biological Anthropology, Natural History Museum, Cleveland, OH USA; 4 Department of Neurosurgery, Clinical Neuroscience Research Center, Tulane University School of Medicine, New Orleans, LA USA; 5 Department of Oral Pathology and Medicine, Faculty of Medicine, Dentistry and Pharmaceutical Sciences, Okayama University, Okayama, Japan; 6 Department of Otorhinolaryngology, Graduate School of Medical Sciences, Kyushu University, Fukuoka, Japan; 7 Department of Neurology, Clinical Neuroscience Research Center, Tulane University School of Medicine, New Orleans, LA USA; 8 Department of Structural & Cellular Biology, Tulane University School of Medicine, New Orleans, LA USA; 9 Department of Neurosurgery and Ochsner Neuroscience Institute, Ochsner Health System, New Orleans, LA USA; 10 Dental and Oral Medical Center, Kurume University School of Medicine, 67 Asahi-Machi, Kurume, Fukuoka Japan; 11 Division of Gross and Clinical Anatomy, Department of Anatomy, Kurume University School of Medicine, 67 Asahi-Machi, Kurume, Fukuoka Japan; 12 Department of Anatomical Sciences, St. George’s University, St. George’s, Grenada; 13 Department of Otolaryngology, Tulane University School of Medicine, New Orleans, LA USA; 14 Department of Surgery, Tulane University School of Medicine, New Orleans, LA USA; 15 University of Brisbane, Brisbane, Australia; 16 Department of Neurosurgery, Tulane University School of Medicine, 131 S. Robertson St. Suite 1300, New Orleans, LA 70112 USA

**Keywords:** Skull, Cranium, Defect, Calvaria, Bone, Congenital, Anatomy

## Abstract

**Introduction:**

Enlarged parietal foramina (EPF) are rare, symmetric calvarial defects. Although typically benign, they are clinically significant due to possible associations with venous malformations and inherited genetic variants. However, the anatomical, radiological, and histological details of these entities are scant in the medical literature.

**Methods:**

Two hundred and fifty adult human skulls from multiple anatomical collections were examined for EPF. Gross morphometric data were supplemented by high-resolution microcomputed tomography (microCT) and histological analysis (H&E, PAS, and Masson’s trichrome). Morphology, cortical continuity, and edge characteristics were evaluated to distinguish developmental defects from acquired bone lesions.

**Results:**

Bilateral EPF were identified in two (0.8%) specimens. MicroCT revealed smooth, corticated margins without lytic or reactive changes. Histological sections demonstrated abrupt cortical thinning and fibrovascular connective tissue at the defect edge, without evidence of osteoclastic activity, inflammation, or abnormal deposition. Surrounding bone exhibited normal cortical and trabecular architecture.

**Conclusion:**

Our findings support the theory that EPF represents a localized congenital ossification defect of the parietal bone rather than a pathologic erosion. Their developmental origin likely reflects aberrant parietal notch closure and persistent falcine venous structures. Understanding their embryologic and genetic basis enhances clinical recognition, guides genetic counseling, and informs surgical management strategies.

## Introduction

Enlarged parietal foramina (EPF), classically identified as symmetric, oval, or round, are located near the junction of the sagittal and lambdoid sutures. Typically ovoid or round, the radiolucencies observed in today’s radiological studies were first noted in 1707 in skulls displayed in anatomical museums [1]. EPF was documented in 1772 by Lobstein and received increasing attention throughout the 1940 s [2]. Among the catalog of reports, this anomaly has also been referred to as fenestrae parietales symmetricae, foramina partietalia permagna, and giant parietal foramina [2].

These unusual foramina have since been elaborated upon, and the underlying mechanism and epidemiology further elucidated. During normal fetal development, these bilateral defects of the calvaria are usually present until the fifth month of gestation. The intramembranous ossification that allows for the closure of these passageways is impaired in EPF due to a genetically governed pathway. This ossification defect is specific to the posterior aspect of the parietal bones; when there is insufficient ossification in the vascularized membrane, patients are left with EPF. The incidence of EPF is reported to be 1 in 15,000 to 1 in 50,000, and the condition is usually found in individuals with a family history significant for EPF. It is inherited in an autosomal dominant pattern with incomplete penetrance [2]. The diameter of these foramina varies, generally ranging from a few millimeters to several centimeters [2]. This dimensional spectrum distinguishes EPF from a similar condition, small parietal foramina (SPF), which includes a foraminal diameter between 1 and 2 mm with either unilateral or bilateral defects [2].

In the clinical realm, EPFs are mostly benign but can be associated with other developmental anomalies, including cerebrovascular and craniofacial malformations [3]. One comorbidity of cerebral developmental origin is drainage of the deep cerebral venous system to the sagittal sinus via a persistent falcine plexus [3, 4]. Although most patients are asymptomatic, some can experience epileptic seizures, headaches, nausea, vomiting, or localized pain when pressure is applied. In addition to the spectrum of physical symptoms, EPF is also associated with an increased risk of brain injury secondary to traumatic causes or to complications from surgical procedures.

As the anatomical, radiological, and histological details of these entities are scant in the medical literature, the following study was performed to shed more light on EPF.

## Methods

Two hundred and fifty adult human skulls were examined for EPF. One hundred specimens were derived from the teaching collection of the Tulane University School of Medicine, New Orleans, LA, USA, and 150 from the Hamann-Todd skeletal collection located at the Cleveland Museum of Natural History, Cleveland, OH, USA. The 150 skulls from the TH collection had known ethnicity, sex, and age, while the others, aside from being adults, had unknown ethnicity, sex, and age. A nondecalcified specimen of the parietal bone encompassing the foramen was imaged using high-resolution microcomputed tomography (microCT) (Quantum GX2, Micro CT, PerkinElmer, Waltham, Massachusetts, USA). The exposure volume was set at 36-mm diameter and 36-mm height. MicroCT scans were obtained with a voxel size of 0.144 mm, the X-ray source settings being adjusted to optimize bone-to-soft tissue contrast. The scan was set at 90 kV and 88 µA. Reconstructed datasets were visualized in multiple planes (axial, coronal, and sagittal) to assess cortical and trabecular bone morphology. Three-dimensional volume renderings were generated using standard reconstruction software (OsiriX MD, Pixmeo SARL, Bernex, Switzerland) to evaluate surface morphology, cortical continuity, and defect margins. Cortical thickness, trabecular patterning, and the morphology of the foramen edges were assessed qualitatively. Image analysis focused on differentiating congenital ossification defects from acquired erosive or lytic processes. Two parietal bone specimens containing the EPF were harvested, fixed in 10% neutral buffered formalin, and subsequently decalcified in a formic acid–based solution until they were soft enough for sectioning. Following decalcification, the samples were processed through graded alcohols, cleared in xylene, and embedded in paraffin. Coronal sections were cut at 5–7 μm thickness using a rotary microtome. Routine hematoxylin and eosin (H&E) staining was performed to assess general morphology. Additional special stains included Masson’s trichrome (MT) to reveal collagen deposition and connective tissue organization and periodic acid–Schiff (PAS) to highlight basement membranes and carbohydrate-rich structures. Slides were examined under light microscopy at magnifications ranging from × 40 to × 400. Representative fields were digitally imaged for analysis.

The authors state that every effort was made to follow all local and international ethical guidelines and laws that pertain to the use of human cadaveric donors and their images in anatomical research [5–7].

## Results

### Gross findings

**Fig. 1:**
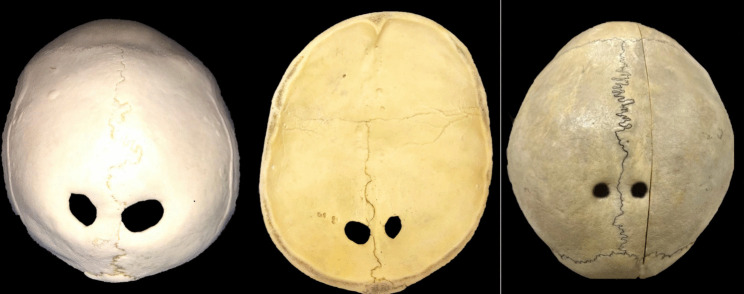
Examples of enlarged parietal foramina. Left and middle images are of the same specimen, showing the external and internal features, respectively. Note that all are near the adjacent sagittal suture. The left and middle images and their foramina have an oval shape, and the right image has foramina that are circular in shape

Bilateral giant parietal foramina were identified in two (0.8%) specimens (Fig. 1). On average, they measured 3.2 cm in diameter (range 2–4.3 cm). The mean distance from the midline to the medial edge of a giant parietal foramen was 3.4 cm (range 0–2.3 cm). The margins of all giant parietal foramina were smooth and beveled.

### MicroCT findings

**Fig. 2:**
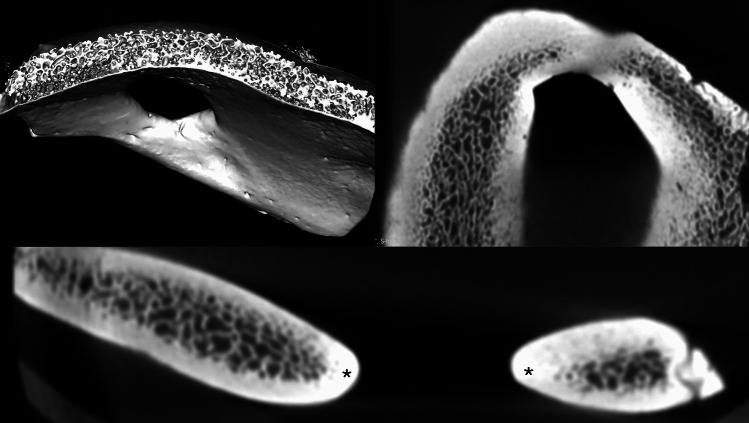
MicroCT analysis of the enlarged parietal foramina demonstrates symmetric, corticated, oval transosseous channels traversing the parietal bones with preserved cortical and trabecular architecture, consistent with congenital parietal foramina rather than acquired defects. The imaging shows clear separation of inner and outer tables and normal diploic morphology, confirming a developmental rather than pathologic origin. The normal but adjacent trabecular patterns can be seen in the volume-rendered image (upper left image), and the cortical rim of the foramen in axial (upper right image) and sagittal images (bottom image). Asterisks, cortical edges of the foramen

Cross-sectional microCT slices showed cortical thinning and interruption at the site of the EPF (Fig. 2). The diploic (trabecular) bone was preserved in adjacent regions but tapered at the foraminal edge, leaving only a thin cortical rim. There was no evidence of cortical erosion, periosteal reaction, or lytic destruction. 3D reconstructions and sectional images showed smooth, corticated, well-demarcated borders around the foramina (Fig. 2). The absence of irregular, moth-eaten, or destructive borders ruled out acquired osteolytic processes such as neoplasm or infection. Volume-rendered microCT demonstrated a round to oval opening extending through the full thickness of the parietal bone. Both inner and outer tables were intact at the margins and merged smoothly into the defect (Fig. 2). No reactive sclerosis was present. Trabecular density and architecture in the surrounding parietal bone were preserved, with no signs of osteopenia, defective mineralization, or systemic bone disease.

### Histology

#### Free edge of the parietal foramina

**Fig. 3:**
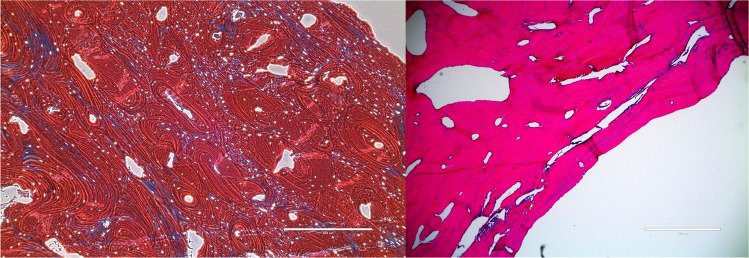
Comparison of normal parietal bone (left; Masson’s trichrome × 40) and the abnormal parietal bone in a decalcified section through an enlarged parietal foramen (right; H&E × 200). Note the abrupt transition between normal parietal bone (upper left half of the left image) and hypoplastic bone at the foramen edge, with loss of marrow space (lower right half of the left image)

At low power, the foramen border showed abrupt cortical thinning with delicate PAS-positive connective tissue bridging the defect. High power revealed fibrovascular connective tissue containing normal vascular basement membranes with no abnormal glycogen or mucopolysaccharide accumulation. These findings exclude metabolic storage disorders. High magnification showed an irregular and scalloped bone edge (Fig. 3). There were few osteocytes toward the margin, and marrow extension was absent. The margins of the foramina contained some osteoclasts and osteoblasts. No osteoclastic resorption, inflammation, or neoplastic changes were identified.

#### Away from the free edge of the enlarged parietal foramina

**Fig. 4:**
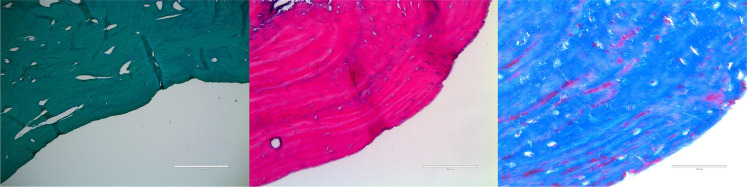
Left: periodic acid–Schiff (PAS) stain demonstrating the sharply defined cortical margin and underlying compact lamellar bone with well-organized osteocytes and limited periosteal irregularity. Center: hematoxylin and eosin (H&E) stain showing viable lamellar bone with osteocytes within lacunae and absence of inflammatory or resorptive changes, confirming a nonpathologic cortical architecture. Right: Masson’s trichrome stain highlighting dense collagenous organization (blue) of the periosteal surface and vascular channels near the foraminal rim (red), consistent with normal remodeling and vascular adaptation rather than erosion. Scale bars = 200 µm (left, middle) and 100 µm (right)

The surrounding parietal bone was histologically normal. Sections distant from the margin demonstrated normal cortical and trabecular bone with intact marrow spaces. PAS highlighted typical vascular and stromal elements without abnormal deposition. This confirmed that these foramina are localized and not representative of diffuse bone disease. The margins of the foramina showed hypoplastic cortical bone, diminished marrow extension, and replacement by normal fibrovascular connective tissue (Fig. 4). There was no evidence of osteolysis, inflammation, or abnormal deposition. No signs of metabolic or neoplastic disease were identified.

## Discussion

The findings of our study, confirmed by histological analysis and microCT, support the view that giant parietal foramina result from a developmental defect in membranous bone ossification and are not due to disease processes such as secondary erosion. The foramina all had well-circumscribed, corticated margins with a smooth transition of cortical surfaces into the defects. The surrounding parietal bone was normal grossly, histologically, and per imaging.

The underlying defect leading to EPF can be better understood in the context of normal neural development. Embryological development of the cerebral venous system includes the development of the inferior aspect of the sagittal sinus, which is believed to originate from the ventral aspect of the sagittal plexus following the disappearance of smaller channels within the plexus. The current consensus endorses the view that the posterior third of the plexus contains multiple channels of the sagittal plexus, ultimately contributing to the development of the superior sagittal sinus. A 2007 study of the anatomy of the falcine plexus concluded that all falcine venous plexuses communicated with the inferior sagittal sinus, but only 63% communicated with the superior sagittal sinus [8]. The falcine sinus is present within the falx cerebri throughout embryological development, and is ultimately obliterated before birth [4, 8]. Its persistence after birth, an anomaly known as persistent falcine sinus, is associated with various cranial abnormalities, including bilateral EPF [8].

Normal embryological development also includes ossification of the parietal bones, beginning around the 7th or 8th week of gestation. The straight sinus develops from the primitive venous plexuses by week 10 [9]. Developmental structures such as neural crest cells and the paraxial mesoderm are directly implicated in brain development and the ossification of the surrounding parietal bones. A small line of neural crest cells remains between the two parietal bones, contributing to signaling and growth and also assisting development of the underlying meninges [10]. Abnormal processes within any of these aspects of development can contribute to abnormal and incomplete formation of the local venous system [10].

In addition to interruptions of venous growth, insufficient or defective ossification around the parietal notch leads to the enlarged foramina characteristic of EPF. Such defects can be found in the upper posterior angle of parietal bones close to the intersection of the sagittal and lambdoid sutures, between the posterior and middle thirds of the sagittal suture [2, 11].

Along with associated abnormalities, most EPF patients also have a positive family history. The condition is inherited in an autosomal dominant fashion with incomplete but high penetrance. Specific mutations in the MSX2 or ALX4 genes are associated with EPF, identified by sequence analysis testing such as genomic hybridization and whole gene exome deletion testing [2]. Given the inheritance patterns and the genes commonly associated with EPF, patients are encouraged to participate in genetic counseling. Prenatal diagnosis and preimplantation genetic testing at 18 to 20 weeks of gestation are recommended for families with a known genetic defect associated with EPF [2, 12].

Regarding clinical manifestations and diagnosis, enlargement of parietal foramina can be identified in utero using neonatal ultrasound. Other diagnoses are discovered incidentally later in life on MRI, CT, or by palpation on physical examination [13]. Following a diagnosis of EPF, management includes treatment of specific symptoms if they arise. It also entails educating patients and their families on safety precautions, including the increased risk of brain injury owing to the exposure of brain parenchyma.

Although they are often clinically asymptomatic, the symmetric ovals at the interface between the sagittal and lambdoid sutures present a unique problem in neurosurgery. When indicated, surgical intervention involves correction of the foramina and reinforcement of surrounding areas via autologous calvarial bone graft or hydroxyapatite plates. Cranioplasty can mitigate the potential for traumatic brain injury in these patients, but it presents its own risks. As foreign material is introduced to the patients’ parenchyma, autologous graft rejection is always a concern [14]. As with any neurosurgical procedure, there is also the risk of perioperative infection.

Additionally, correction of EPF is often recommended in infants, a population already predisposed to infection secondary to immaturity of the immune system. Complications associated with cranioplasty in the pediatric population are often studied following initial craniectomy. Similar perioperative concerns can be applied to infants receiving cranioplasty secondary to EPF and include bone resorption, plagiocephaly, and underlying subdural fluid collection [15]. Additional studies of neurosurgical repair of EPF could further elucidate the risks and complications specific to this unique patient population.

## Conclusion

Our findings reinforce that EPF are a congenital manifestation of impaired membranous ossification rather than a consequence of acquired osteolytic, metabolic, or inflammatory disease. Through integration of gross morphology, microCT imaging, and histological analysis, this study demonstrates that the margins of EPF are corticated, smooth, and structurally continuous with adjacent normal bone, and that the surrounding parietal tissue retains intact trabecular architecture and marrow composition. These features confirm a localized developmental defect rather than systemic bone pathology.

Clinically, EPF is usually asymptomatic but warrants awareness due to its association with venous and craniofacial anomalies and the potential for traumatic brain injury. MicroCT and histologic correlation provide reliable differentiation of EPF from pathologic skull lesions, improving diagnostic accuracy in radiologic and surgical settings. Future studies incorporating genetic analysis and pediatric imaging correlation may further delineate the developmental continuum between small and giant parietal foramina and refine recommendations for surgical repair.

## Data Availability

No datasets were generated or analysed during the current study.

## References

[1] Halbertsma T (1940) Fenestrae parietales symmetricae. Arch Dis Child 15:115–120 21032167 10.1136/adc.15.82.115 PMC1987727

[2] Griessenauer CJ, Veith P, Mortazavi MM, Stewart C, Grochowsky A, Loukas M, Tubbs RS (2013) Enlarged parietal foramina: a review of genetics, prognosis, radiology, and treatment. Childs Nerv Syst 29(4):543–547. 10.1007/s00381-012-1982-7 23207976 10.1007/s00381-012-1982-7

[3] Mavrogiannis LA, Wilkie AOM (2024) Enlarged parietal foramina. [Updated 2019 Nov 27]. In: Adam MP, Mirzaa GM, Pagon RA, et al. (eds) GeneReviews® [Internet]. University of Washington, Seattle (WA). Available from: https://www.ncbi.nlm.nih.gov/books/NBK1128/. Accessed 15 Aug 2025 20301307

[4] Choudhary Arabinda K, Smith A, Smith A, Choudhary AK (2014) Prevalence of persistent falcine sinus as an incidental finding in the pediatric population. AJR Am J Roentgenol 203(2):424–5. 10.2214/AJR.13.10799 25055280 10.2214/AJR.13.10799

[5] Iwanaga J, Kim HJ, Akita K, Logan BM, Hutchings RT, Ottone N, Nonaka Y, Anand M, Burns D, Singh V, Peris-Celda M, Martinez-Soriano F, Apaydin N, Hanna A, Yoshioka N, Fernandez-Miranda J, Hur MS, Shoja MM, Saremi F, Reina F, Tabira Y, Carrera A, Spratt JD, Ho SY, Mori S, Komune N, Watanabe K, Prats-Galino A, De Andrés J, Reina MA, Abrahams PH, Anderson RH, Ibaragi S, Loukas M, Tubbs RS (2025) Ethical use of cadaveric images in anatomical textbooks, atlases, and journals: a consensus response from authors and editors. Clin Anat 38(2):222–225. 10.1002/ca.24258 39754475 10.1002/ca.24258

[6] Iwanaga J, Singh V, Ohtsuka A, Hwang Y, Kim HJ, Moryś J, Ravi KS, Ribatti D, Trainor PA, Sañudo JR, Apaydin N, Şengül G, Albertine KH, Walocha JA, Loukas M, Duparc F, Paulsen F, Del Sol M, Adds P, Hegazy A, Tubbs RS (2021) Acknowledging the use of human cadaveric tissues in research papers: recommendations from anatomical journal editors. Clin Anat 34(1):2–4. 10.1002/ca.23671 32808702 10.1002/ca.23671

[7] Iwanaga J, Singh V, Takeda S, Ogeng’o J, Kim HJ, Moryś J, Ravi KS, Ribatti D, Trainor PA, Sañudo JR, Apaydin N, Sharma A, Smith HF, Walocha JA, Hegazy AMS, Duparc F, Paulsen F, Del Sol M, Adds P, Louryan S, Fazan VPS, Boddeti RK, Tubbs RS (2022) Standardized statement for the ethical use of human cadaveric tissues in anatomy research papers: Recommendations from Anatomical Journal Editors-in-Chief. Clin Anat 35(4):526–528. 10.1002/ca.23849 35218594 10.1002/ca.23849

[8] Tubbs RS, Loukas M, Louis RG Jr, Shoja MM, Acakpo-Satchivi L, Blount JP, Salter EG, Oakes WJ, Wellons JC 3rd (2007) Anatomy of the falcine venous plexus. J Neurosurg 107(1):155–7. 10.3171/JNS-07/07/0155 10.3171/JNS-07/07/0155 17639885

[9] Fink AM, Maixner W. Enlarged parietal foramina: MR imaging features in the fetus and neonate. AJNR Am J Neuroradiol. 2006;27(6):1379–81. PMID: 16775301; PMCID: PMC8133951. PMC8133951 16775301

[10] Jin SW, Sim KB, Kim SD (2016) Development and growth of the normal cranial vault: an embryologic review. J Korean Neurosurg Soc 59(3):192–196. 10.3340/jkns.2016.59.3.192 27226848 10.3340/jkns.2016.59.3.192 PMC4877539

[11] Durão C, Carpinteiro D, Pedrosa F, Machado MP, Cunha E (2016) Enlarged parietal foramina: a rare forensic autopsy finding. Int J Legal Med 130(3):855–7. 10.1007/s00414-015-1239-6 26233611 10.1007/s00414-015-1239-6

[12] Wilkie AOM, Mavrogiannis LA (1993) Enlarged parietal foramina/cranium bifidum. In: Pagon RA, Bird TD, Dolan CR, Stephens K, Adam MP (eds) GeneReviews™ [Internet]. Seattle (WA), University of Washington 20301307

[13] Tubbs RS, Smyth MD, Oakes WJ (2003) Parietal foramina are not synonymous with giant parietal foramina. Pediatr Neurosurg 39:216–217 12944704 10.1159/000072475

[14] Kortesis B, Richards T, David L, Glazier S, Argenta L (2003) Surgical management of foramina parietalia permagna. J Craniofac Surg 14(4):538–44 12867871 10.1097/00001665-200307000-00028

[15] Behbahani M, Rosenberg DM, Rosinski CL, Chaudhry NS, Nikas D (2019) Cranioplasty in infants less than 24 months of age: a retrospective case review of pitfalls, outcomes, and complications. World Neurosurg 132:e479–e486. 10.1016/j.wneu.2019.08.106 31465852 10.1016/j.wneu.2019.08.106

